# Type II NKT cells differentially regulate CD4 T cell subsets

**DOI:** 10.1186/2051-1426-2-S3-P233

**Published:** 2014-11-06

**Authors:** Faith C Robertson, Xiuju Lu, Masaki Terabe1, Jay A Berzofsky

**Affiliations:** 1 Vaccine Branch, Center for Cancer Research, National Cancer Institute, National Institutes of Health, Bethesda, MD, USA; 2 Division of Target Discovery, Otsuka Maryland Medicinal Laboratory, Rockville, MD, USA

## 

Natural killer T (NKT) cells lie at the interface between the innate and adaptive immune systems and are important mediators of tumor immunosurveillance. This CD1d-restricted lymphoid population recognizes lipid antigen and can rapidly produce an array of cytokines and chemokines to modulate the host immune response. In tumor immunity, two NKT cell subsets (type I and type II) have contrasting roles in which they not only distinctly impact innate and adaptive immune cell populations, but also cross-regulate one another. Type I NKT cells are usually associated with the promotion of tumor immunity whereas type II NKT cells seem to suppress it. Previous studies showed that type II NKT cells negatively impact CD8^+ ^T cells; however, little is known about their effect on CD4^+ ^T helper subsets. In this study, we investigated how CD4^+ ^T cell subsets are affected by type II NKT cells activated with sulfatide, an endogenous glycolipid antigen known to activate type II NKT cells. Naïve ovalbumin (OVA) specific CD4^+ ^T cells isolated from DO11.10 transgenic mice were either tested *ex vivo*, or polarized *in vitro *into T helper subsets (Th1, Th2 and Th17). CFSE dilution and ELISAs were used to assess CD4^+ ^T cell proliferative and cytokine responses upon antigenic stimulation in the presence or absence of sulfatide-activated type II NKT cells. We found that activated type II NKT cells significantly inhibited proliferation of naïve CD4^+ ^T cells. The differentiated activated/memory subsets appeared to be less susceptible to such suppression. Ongoing elucidation of these interactions and mechanisms may not only augment our understanding of CD4^+ ^T cell regulation, but also improve our capability to utilize type II NKT cells for clinical applications.

